# Supportive Communities: Conceptualizing Supportive Structures for Coaches’ Learning and Well-Being in Community Youth Soccer

**DOI:** 10.3390/ijerph19148249

**Published:** 2022-07-06

**Authors:** Krister Hertting, Karin Grahn, Stefan Wagnsson

**Affiliations:** 1 School of Health and Welfare, Halmstad University, 30118 Halmstad, Sweden; 2 Department of Food and Nutrition and Sport Science, University of Gothenburg, 40530 Gothenburg, Sweden; karin.grahn@ped.gu.se; 3 Department of Educational Studies, Karlstad University, 65188 Karlstad, Sweden; stefan.wagnsson@kau.se

**Keywords:** coaching, voluntary coaches, health, well-being, community of practice, youth sports, soccer, learning

## Abstract

Sweden has an extensive culture of community club sports for children and youths, based on voluntary leadership. Being a voluntary coach can be stressful and can affect coaches’ well-being. Since voluntary coaching is closely connected to and conditioned by family life and civil occupation, coaches need support for practical issues as well as for developing their coaching assignment in relation to the constantly changing conditions within sports for children and youths. The aim of this paper was to conceptualize and problematize the supportive structures in everyday activities, in order to promote learning and well-being and to prevent mental health issues. This paper is conceptual and based on a paradigm case. The starting point is communities of practice (CoP) and how CoPs can contribute to the development of supportive structures for coaches in youth sports. In conclusion, a CoP is dependent on negotiation within the coaching team as well as on facilitating factors that can add knowledge, perspectives, and experiences to the CoP. The facilitating factors mean that a CoP has the potential to be health-promoting for both coaches and players. Hence, it is important to create conditions, structures, and support—such as policies, practice-based education, mentorship, and facilitators.

## 1. Introduction

When a group of people who have common interests and share specific areas of knowledge and competence meet, several opportunities for development and mutual learning arise through discussions on how to create the best practice. Organized sports for children and youths provide a central arena where such meetings and discussions are ongoing. Being involved as a coach in community sports for children and youths is a journey, where coaches follow their children for a few years before either the children or coaches drop out or continue elsewhere. The journey is characterized by “learning as you go along” and is connected to coaches’ health and well-being. This is the starting point of the current paper.

Sport coaching has been described as a complex endeavor in an uncertain environment [1]. Being a coach for children and youths can, thus, be stressful and can negatively affect the coaching and learning environment. Surujlal and Nguyen [2] argued that the health and well-being of the coaches are a necessity in educating and inspiring others. In addition, Stebbings, Taylor, and Spray [3] stressed that coaches who experience higher levels of positive outcomes are more likely to trust their athletes’ abilities and to encourage them to pursue empowering possibilities. This is supported by Alcaraz, Torregrosa, and Viladrich [4], and by Thellwell et al. [5], who all suggested that coaches who experience psychological well-being are more likely to develop healthy relationships with their athletes.

Volunteer coaches in children and youth sports are, according to Potts, Didymus, and Kaiseler [6], especially under-researched groups. These coaches complete their assignments in their leisure time and are often a parent of one of the children in the group. In Sweden, sports are largely supported by voluntary labor [7], where almost 25% of the adult population spends a part of their leisure time engaging in more than 20,000 sports associations in the country [8,9]. Hence, in addition to a regular job, coaches in youth sports generally perform the mission in their leisure time, i.e., they are voluntary coaches. In their study, Potts, Didymus, and Kaiseler [6] found stressors among voluntary coaches that were athlete-related (i.e., development vs. competition), coach-related (i.e., balancing multiple roles), and organizational (i.e., parents). In a Swedish study, volunteer soccer coaches considered the demands from their main employment, followed by catching up with the family, and having limited time to plan activities as the most stressful aspects of being a volunteer coach [10]. Since voluntary coaching is closely connected to and conditioned by family life and civil occupation [11], coaches need support for their coaching assignments in relation to the constantly changing conditions within sports for children and youths [10]. In addition, Fletcher and Scott [12] (2010) found that, in general, coaches at the higher levels of competitive sports encounter greater numbers of stressors, compared to the lower-level coaches. Moreover, the demands and the levels of stress among voluntary coaches increases with the age of the players [10]. According to Potts, Didymus, and Kaiseler [6], coping strategies such as social support, time for reflection, and mentorship were central, and it was found that “the art of coping comes with experience, knowledge and understanding” (p. 64).

There are studies that have emphasized the connections between well-being, support, and learning. This is exemplified in the work undertaken by Carson et al. [13] on Australian sports coaches’ mental well-being, which concluded that organizational support and education were crucial for coaches to balance their assignments and well-being.

Furthermore, to support well-being and learning, several studies stress the importance of social support such as supervision and mentoring, as well as structures to allow reflection with other coaches in local club settings [14,15,16,17,18]. Gilbert and Trudel [19] stated that so-called coaching pods could serve as a promoting structure for coaches in community-based sports associations. This means that the “Coaches of teams within similar athlete age groups could form a peer network and discuss coaching issues” (p. 32).

The voluntary coaching assignment can be stressful [6,10] and coaches need support to manage their stress and learning [14,15,16,17,18]. The research points to mentorship as a support strategy [17,18,20]. However, it relies on individuals’ willingness to engage as mentors, and more knowledge is needed on how social supportive structures in everyday activities for voluntary coaches are implemented (c.f. Potts, Didymus, and Kaiseler [6]). Hence, we propose further studies on how to include supportive structures in youth soccer coaches’ everyday practices in sports clubs. The aim of this paper is, therefore, to conceptualize and problematize the supportive structures in everyday activities, for the promotion of learning and well-being as well as for the prevention of mental health issues. The paper is conceptual, with examples drawn from coaching practices. The starting point is a paradigmatic case, (c.f. Alerby and Hertting [21]) focusing on a typical soccer team and their coaches in a community-based club in Sweden. Soccer is the largest sport in Sweden and the Swedish Football Association (SFA) is divided into 21 regional football associations, which locally administer more than 1,000,000 members (players, coaches, leaders, and referees), in almost 2900 clubs [9]. The case is based on previous studies [10,22,23] and our own experiences of coaching. Furthermore, the case will be theoretically informed by Wenger’s [24] concept of communities of practice (CoP) and how CoPs can contribute to coaches’ well-being through the development of supportive learning structures in youth sports.

## 2. The Paradigmatic Case: Small Town FC

Small Town FC is a soccer club in a rural housing area in Sweden. The club has teams for boys and girls and is mainly focused on soccer for all. The club is an important meeting place for the children and youths in the local community. The coaches are mainly parents (mostly fathers) who are doing their coaching voluntarily, in their leisure time. There is a club policy stating that soccer for all should be the focus, but it has been a number of years since it was updated and clearly communicated with the coaches. In addition, the club used to hold courses in which coaches from different teams could learn from each other, and the coaches also participated in courses offered by the SFA. This has declined in recent years and the main reason for this is that there used to be a hired club manager, however, over the last two years (due to the economic situation) the club has been run voluntarily by the board and officials. This has led to a situation where common club activities have decreased and the teams are more separated, with decreased collaboration as a consequence. Occasionally a team has ambitions to compete at a higher level, a decision which nowadays is primarily made by the team rather than by the club. This mainly occurs when the players are entering their teenage years and the games are becoming more competitive.

One of the teams in the club is the Youth 14 team, which has three coaches, all of whom are also parents to players in the team. Kent, Andy, and Steve have been coaching the team together since the children were six years old and started to play soccer. All three of them have a background as soccer players and when the question was posed by the club, they were willing to volunteer as coaches. Kent has played soccer at semi-professional levels, while Andy and Steve have played at grassroot levels. However, all three have a good understanding of the game’s logic and the culture of being on a soccer team. Besides the coaching assignment, they all have families and other children who play soccer, as well as full-time employment. Kent is a carpenter, Andy is a teacher, and Steve is a senior manager in a company. Steve’s child is one of the star players on the team, while the children of Kent and Andy struggle to make it to the first eleven players on the team.

When the coaching team was formed it was decided that Kent should take the lead, since the other coaches considered him to be the most experienced and to have the best competence. They all participated in the initial policy education within the club, and over the years they have participated in weekend courses as part of the SFAs education program, focusing on coaching children and youths. Even though they considered it quite time-consuming, they all participated. However, for the last three years the club has not been able to offer the coaches this education due to the poor economy. It is at present, therefore, only offered to those who are coaching younger children. This means that most of their learning and development as a coaching team in Youth 14 is based on their common experiences and communication within the team.

The group of parents around the team are mostly supportive, which was especially evident in the younger years. However, over the years a group of parents have wanted to focus more on competitiveness, which is something that the coaches need to cope with. As the years have passed, the demands from the club that the coaches participate in activities to raise money have increased. Even though other parents assist in these activities, they all feel that this interferes with their main assignment as coaches.

During their years of coaching, they have been aware of their different ideas and values about how to coach within their coaching team. In the first years, everything was about fun and play. Considering the “soccer for all” policy in the club, Andy always advocated that this should be the guiding idea for the team, even when the players grew older. On the contrary, Steve has always been more focused on the values of performance and competition. Somewhere in between, Kent felt that he could relate to both positions. He was taught in a performance context but has a child with limited soccer competence. In the early years, the coaching team considered the input from the club, other teams, and the SFAs education program as valuable. However, since this input decreased, they feel more as though they are left on their own as a coaching team. Next year the team has a decision to make. They could either try to qualify for the national competition or they could continue to play in the local soccer league.

To summarize the situation, higher demands and different values within the coaching team have become more evident, as have higher demands from a group of parents, family obligations, demands from their work, and having to engage in more activities to raise money. These have all contributed to a higher level of stress. Kent, Andy, and Steve still love their coaching assignment, particularly being with the youths and taking part in their development as players and humans. However, they realize that something needs to be changed in the coaching team. Luckily, the club has realized that the situation is challenging for many of the teams and a new club manager is on the way. One of the main tasks ahead of them is to focus on the development of the club’s culture, as well as on creating healthy coaching environments. Under the current circumstances, the coaches find themselves in a stressful situation.

## 3. A Theoretical Reflection: Constructing Healthy Coaching Communities

In relation to leadership, organizational culture, such as in a sports club, can constitute a strong or weak culture of health [25,26]. Payne, Cluff, and Lang [27] identified that the relational elements of participant engagement, peer support, and leadership support tend to be associated with a culture of health. Meacham, Webb, and Krick [28] defined over twenty elements of a healthy culture that influence organizational health and wellbeing. Among those twenty, there were empowerment, training and learning, relationship development, and a sense of a cohesive community. According to Torp, Eklund, and Thorpenberg [29], the use of empowering settings and participatory approaches are beneficial for health promotion and organizational development. Furthermore, Coenen, Schelfhout, and Hondeghem [30] studied school principals’ professional learning communities and concluded that these supported the principles’ well-being as well as their learning. Based on the section above, we argue that there is a connection between social support and cohesion, well-being, and learning for coaches in sports clubs. In the following two sections, we outline the theoretical basis of the paper.

Influences on youth soccer coaches’ mental health and well-being are multifaceted, with different levels of each aspect influencing it in different scenarios. McLeroy et al. [31] have developed a model for health-related behaviors and health promotion work. McLeroy et al. [31] identified the following five levels of influence: intrapersonal (such as coaching knowledge); interpersonal (interaction with other coaches, players, and parents); institutional (such as club policies and routines); community (national soccer federation and educational programs); and, finally, the policy level (sports policies and laws such as the convention on the rights of the child). To take into consideration the complex practice of coaching [4] and the diverse levels of influences on coaches’ health and well-being [31] we, therefore, argue that Wenger’s [24] communities of practice (CoP) could be a fruitful avenue for developing healthy coaching communities in youth soccer. In this paper, more specifically, we focus on coaching communities of practice (CCoPs), as developed by Culver and Trudel [32].

The foundation of Wenger’s [24] theory of CoP is the social nature of being and learning, where “groups of people who share a concern or a passion for something they do and learn how to do it better as they interact regularly” [33] (p. 1). Through participation in social practices and the negotiation of meaning, people mutually create and are being created by their surroundings. According to Wenger [24,34], a community itself does not automatically constitute a CoP, it contains the following three characteristics: (1) a shared domain of common interests (such as soccer as a global phenomenon); (2) a community where people engage in joint activities, discussions, sharing information, and help each other (such as a soccer club); and (3) a context of practitioners where people develop a shared repertoire of resources, such as experiences, stories, and tools (e.g., a soccer team). Hence, a CoP is based on mutual engagement, joint enterprise, and a shared repertoire [24]. Being involved in a CoP is individual and closely connected to identity, but the formation of a CoP is also a negotiation of identities [24,34]. Therefore, a CoP always consists of active negotiation and engagement with the tensions and challenges between participants. In a soccer context, for instance, negotiation about ambitions and goal setting for the team, how to prepare for the season, and which exercises to include in training could be subjects for negotiation and tensions [32]. However, coaches are often involved in broader social learning systems and other CoPs that will affect the coaching assignment [31]. Finally, Wenger [34] stresses the meaning of boundary objects, which also support the connections between CoPs. Boundary objects can be artifacts (tools, documents, and models), discourses (common language), and shared processes (routines and procedures). In summary, and in the case of soccer coaches, Culver and Trudel [32] stated that “When coaches sustain mutual engagement in their enterprise long enough to share significant learning, a CCoP is the result.” (p. 100).

Previous studies on cultivating and maintaining CoPs in sports coaching practices have shown the importance of having a structure as well as the important role of a facilitator in the group for learning to occur [35]. In a study of a CoP within a group of Alpine skiing coaches, Garner and Hill [36] showed both interpersonal and intrapersonal learning within the CoP. Interpersonal learning was demonstrated through emotional intelligence and storytelling, resulting in a more athlete-centered approach to coaching practice. Intrapersonal knowledge was developed through group reflections, which changed their views on the coaches’ own professional roles. Furthermore, Bertram, Culver, and Gilbert [37] argued that CoPs can support learning and development in the sports coaching setting. As an example, coaches learned about other topics than those in the formal coaching education and they had an opportunity to address matters that were of relevance to their ongoing coaching practice.

## 4. Reflecting on Smalltown FC and the Youth 14 Team as a CCoP

Using the terminology of Wenger [24,34], Smalltown FC could be seen as a community of people with shared interests and joint activities within a shared domain-soccer. Following this, the Youth 14 team could be considered a context in which coaches develop a shared repertoire of resources, experiences, stories, and tools, or, in other words, a CCoP. In this section, the paradigmatic case is highlighted through the lens of a CCoP and is related to coaches’ well-being. Finally, conclusions are drawn, and suggestions for future empirical research are outlined.

### 4.1. CCoP without Influences—Risking Isolation

The CCoP was initiated when the three fathers decided to volunteer as coaches for their children and were well supported by the club. The changing situation in the club, with no employed club manager and coach education only offered to the coaches of younger children, led to less influence from others, and the coaches became more and more reliant on each other. On the one hand, this could lead to stronger cohesion in the coaching team and, consequently, to a stronger CCoP, since continuous discussions that conclude with agreements create a sense of belonging that only strengthens groups [24,34] and creates the conditions for well-being [27,29]. On the other hand, as Culver and Trudel [32] point out, a functioning CCoP is dependent on influences from others. If a limited number of influences are reaching the group, for instance, a lack of input from other coaches or from the club manager, this can enhance stressors such as development vs. competition [9]. For instance, too much focus on competitive values (e.g., not allowing some children to participate in certain games), could be challenging in relation to the rights of children, as set out in the UN convention, which has been regulated in Swedish law since 2020 [38]. Moreover, this way of structuring the team (a high level of competitive sports) goes against the policy of the SSC and the SFA. On an interpersonal level [31], the tension between competitive and equity values heightens the risk of creating stressful situations in the group of coaches and among the parents, as well as between the coaches and parents [6,12].

To provide a hypothetical example from Small Town FC, we can imagine how Kent, who is a former semi-professional soccer player, may use his status as both a former elite player but also as a coach to let his own child play most of the games, despite them being less competent in soccer than other children who are occasionally left out of the team. This may create feelings of frustration and stress within Andy, since he feels that it is unfair to let Kent’s child play more than others who are playing at the same level, which, in the long run, will challenge Andy’s mental well-being, especially, if Andy is holding back his own child in order to be considered a fair coach. Andy has to compromise his own norms, values, and knowledge on an intrapersonal level with the norms, values, and knowledge on an interpersonal level, shaped within the CCoP in the team [31]. Overall, concerns about fairness can lead to stress among coaches, parents, and players. Being in a situation in which you have to balance multiple roles as a coach (and parent) can be stressful [6], especially in a situation that lacks a supportive club policy and common social support from the club [14,19,20]. Indeed, a solution would be to include a coach who is not a parent but, in reality, this is a challenge for many clubs. However, the CCoP would be strengthened by receiving institutional-level support [27,28,31] for revitalizing the club policy document and including it in the CCoPs discussion. The club policy (in its early state) stated that the club should have a “football for all” policy. Since this document has not been activated in recent years, it may need revision and the club coaches could be engaged in the process.

### 4.2. Negotiating Identities, Boundaries, and the Future

As Wenger [24,34] elucidated, choosing to be involved as a coach in a CCoP is closely connected to identity, but participation also means a negotiation of value positions and ideas. In other words, it means negotiating identities as coaches. Developing functioning interpersonal relationships is important for a health-promoting coaching environment [27,28,31]. This is also true in youth sports, where Surujlal and Nguyen [2], Alcaraz, Torregrosa, and Viladrich [4], and Thelwell et al. [5] suggested that those who are healthy and feel good are more likely to develop healthy relationships with their athletes. However, having healthy relationships is fluid and changes over time, and by using the concept of negotiating identities [24] these intra- and interpersonal processes that McLeroy [31] highlights can be elucidated. In Small Town FC, there were ongoing negotiations about identities and ideas within the team. When initiating the CCoP it was negotiated that the coaches’ soccer background was valued more highly than other experiences, given the specific context. In the early years, the boundaries of the club as a community were communicated through club policy, by the club manager, and through common education. As discussed above, over the years the negotiation of boundaries and identities have been more connected to the team or the CCoP [32], and the common community (club) boundaries have decreased in importance. Initial differences in the coaching values became increasingly important in the current situation. Over time, there were complicated negotiations about coaching identities; the limited time available for undertaking voluntary coaching; which value positions should be prominent in the team; and the children growing older, precipitating a shift in focus and values. However, being a voluntary coach and coaching your own children is closely connected to family life [11] and can cause intrapersonal value conflicts. As in the case of Kent, who had strong competitive values on the one hand, but on the other hand, had a child at risk of dropping out because of the overly competitive environment. Hence, being in a voluntary coaching team in youth sports means balancing intrapersonal values, relational elements, and value positions [28,29]. So, in Small Town FC, one crucial negotiation was about which values and boundaries would apply in the future. For long-term well-being in the assignment as a coach, we argue that the coaching identity needs to be fairly matched with the basic ideas of the coaching assignment over time. This aligns with ideas of authentic leadership, and Weiss et al. [39] argued that authentic leadership facilitates leaders’ stress reduction, increases commitment, and maintains their mental well-being.

### 4.3. Changing and Dynamic Communities

A community-based soccer club such as Small Town FC, with a long history, many teams, and an important meeting place for people, can appear as something that will be almost eternally present. However, change is inevitable and constant on different levels. The shared domain of soccer is affected by societal changes. As an example, the UN convention on the rights of the child being regulated by law in Sweden is one such change at the policy level [31]. This affects the national governing bodies (NGOs) in sports, such as the SFA and the SSC, causing them to develop their policies. Furthermore, at the community and institutional level [31] sports clubs and coaches are affected by these changes through the demands communicated by the NGOs to the clubs and associations and through coaching education. In the case of Small Town FC, coaches need to take the children’s rights into consideration when planning and implementing soccer practice. However, the question of how to incorporate the perspective of children’s rights into practice is not adequately communicated at the policy and community level, leaving it up to the coaches to find solutions on how to practice sports according to the UN convention on the rights of the child. It might be stressful for individual coaches not to know how to incorporate this into practice. As already shown in the example of Small Town FC, this may cause tensions in the CCoP. While Andy stresses the importance of sports for all, Kent and Steve push to focus the team on more competitive values. To release the tension and to manage the mission to incorporate the perspective of children’s rights into practice, the coaches in Small Town FC need education and support. Such support is, according to research by Carson et al. [13], essential for coaches’ well-being. One way to create such support for coaches is to formalize a CCoP, where discussions of common issues within the Small Town FC can take place, in order to seek support from other coaches and teams within the club [35,36]. In this process, boundary objects, such as tools, documents, or models, and a common language for child-centered coaching in Small Town FC’s teams could be part of the development [34].

Other changes at the institutional level [31] may affect coaches in youth sports. In connection to the case of Small Town FC, prerequisites in the club are changing. From being a club with clearly communicated boundaries (on a policy level), common courses for coaches from different teams, and a club manager as “the spider in the net”, to a community characterized by the negotiation of boundaries and by the concept of “every team for themselves” has profoundly changed the dynamics of the club community. In line with these changes, one can imagine a similar change in the CCoP, from a more club-based community to a more team-based community. Furthermore, the dynamics in the parent community surrounding the team and the activities for raising money have changed and have increased the pressure on the coaches. This may cause stress for the coaches in charge [10]. Finally, the conditions of soccer are changing continuously as the players grow older, which raises the pressure on coaches [10]. Overall, the dynamics of change are constantly present, affecting coaches in their everyday practices [1], and if the coaches in Small Town FC should have the possibility to develop their identities and a CCoP they need to be in phase with these changes. One path might be to include supervision and mentoring in the club [14,20] to support coaches in these changes.

## 5. Conclusions

This paradigmatic case study was an attempt to illuminate how voluntary coaching can be expressed in the context of youth soccer, and to conceptualize and problematize CCoPs as supportive structures, which promote coaches’ learning and well-being and prevent ill-health issues. In conclusion, the case showed that a CCoP is dependent on negotiation within the coaching team, as well as on facilitating factors that can add knowledge, perspectives, and experiences to the CCoP. Through interactions between the coaching team and the facilitating factors, favorable conditions for a well-functioning CCoP, which has the potential to be health-promoting for both the coaches and the players, can be achieved. Hence, it is important to create conditions, structures, and support, such as policies, practice-based education, mentorship, and facilitators (for instance a club manager). Empirical research needs to focus on how the concept of CCoPs can be applied and developed in youth sports. When studying CCoPs in youth-sports settings, issues on gender, equality, and the rights of children should be taken into consideration.

## Data Availability

Not applicable.
